# A Study on the Adsorption of Rhodamine B onto Adsorbents Prepared from Low-Carbon Fossils: Kinetic, Isotherm, and Thermodynamic Analyses

**DOI:** 10.3390/molecules29061412

**Published:** 2024-03-21

**Authors:** Aleksandra Bazan-Wozniak, Aleksandra Jędrzejczak, Robert Wolski, Sławomir Kaczmarek, Agnieszka Nosal-Wiercińska, Judyta Cielecka-Piontek, Sultan Yagmur-Kabas, Robert Pietrzak

**Affiliations:** 1 Department of Applied Chemistry, Faculty of Chemistry, Adam Mickiewicz University in Poznań, Uniwersytetu Poznańskiego 8, 61-614 Poznań, Poland; aleksandra.bazan@amu.edu.pl (A.B.-W.); alejed6@st.amu.edu.pl (A.J.); robert.wolski@amu.edu.pl (R.W.); slawomir.kaczmarek@amu.edu.pl (S.K.); 2 Department of Analytical Chemistry, Faculty of Chemistry, Institute of Chemical Sciences, Maria Curie-Skłodowska University in Lublin, Maria Curie-Skłodowska Sq, 3, 20-031 Lublin, Poland; agnieszka.nosal-wiercinska@mail.umcs.pl; 3 Department of Pharmacognosy, Faculty of Pharmacy, Poznan University of Medical Sciences, Rokietnicka 3, 60-806 Poznań, Poland; jpiontek@ump.edu.pl; 4 Department of Chemistry and Chemical Processing Technology Programs, Lapseki Vocational School, Çanak-kale Onsekiz Mart University, 17800 Çanakkale, Türkiye; syagmur@comu.edu.tr

**Keywords:** activated carbon, chemical activation, kinetic, isotherm study, thermodynamic study, rhodamine B

## Abstract

The aim of this study was to obtain a series of activated carbon samples by the chemical activation of low-rank coal. The precursor was impregnated with a NaOH solution. Activated carbons were characterized by determining their textural parameters and content of surface oxygen functional groups and by using an elemental analysis. The carbons were tested as potential adsorbents for the removal of liquid pollutants represented by rhodamine B. The effectiveness of rhodamine B removal from water solutions depended on the initial concentration of the dye, the mass of rhodamine B, and the pH and temperature of the reaction. The isotherm examination followed the Langmuir isotherm model. The maximum adsorption capacity of the rhodamine B was 119 mg/g. The kinetic investigation favored the pseudo-second-order model, indicating a chemisorption mechanism. The thermodynamic assessment indicated spontaneous and endothermic adsorption, with decreased randomness at the solid–liquid interface. The experiment revealed that a 0.1 M HCl solution was the most effective regenerative agent.

## 1. Introduction

Adsorption is a phenomenon that occurs on the surface of a solid [1]. It involves the attachment of adsorbate molecules to the surface of the adsorbent. Activated carbons are frequently utilized for adsorbing gaseous and liquid pollutants, allowing for the elimination of organic and inorganic pollutants from water, wastewater, and air [2,3,4,5]. The porous structure of activated carbons is primarily responsible for their high adsorption capacity, enabling an efficient adsorption process. It is important to note that the chemical structure of the adsorbent allows chemical reactions to take place between the functional groups of the carbon and the adsorbate to be removed. The efficiency of the adsorption process is influenced by various factors, including the chemical structure of the adsorbent and the parameters of the adsorbate [6,7]. For instance, in the case of the adsorption of aqueous solutions, the concentration, mass, and pH of the adsorbate play a crucial role [8].

Industrial wastewater pollutants pose a significant threat to drinking water. Wastewater contains a vast array of organic substances, including dyes, suspended solids, and other soluble materials. Rhodamine B is a red alkaline dye that belongs to the xanthene group. It is commonly used in the textile and food industries, as well as in the production of pens, paints, inks, and stamp inks, due to its high stability, good water solubility, and non-biodegradability. Rhodamine B enters surface water with wastewater, posing a serious threat to the environment and negatively affecting both human and animal health. The use of rhodamine dye can cause irritation to the eyes, skin, and respiratory system, and can also damage the nervous system and have carcinogenic effects. Ongoing research is being conducted to develop new techniques for treating wastewater containing rhodamine [9,10,11,12].

Chemical activation is one method of obtaining activated carbons [13]. This process allows for the production of adsorbents with a well-developed porous structure and a large specific surface area. It also permits the use of lower temperatures and shorter activation times compared to physical activation [14]. Carbon adsorbents can be prepared using conventional heating (tube furnace) or microwave heating (microwave furnace) [15].

This study offers a thorough examination of the synthesis, physicochemical properties, and adsorption capabilities of activated carbons derived from low-rank coals sourced from the collieries of Labin and Spitsbergen. Key parameters such as the contact time, initial rhodamine B concentration, adsorbent dosage, shaking speed, and temperature were investigated to assess their influence on dye adsorption. The adsorption process was meticulously analyzed, the feasibility of reusing carbon adsorbents was explored, and insights were gained into the underlying mechanisms involved. What is new in our work is the use of sodium hydroxide as an activator in our precursor studies. Our previous studies showed that the use of another activator, potassium hydroxide, favors the obtaining of carbon adsorbents with a high efficiency for the removal of rhodamine B from aqueous solutions [16]. The aim of the present work was to finally determine which of these activators leads to a better adsorption performance.

## 2. Results and Discussion

### 2.1. Characterization of the Activated Carbons

Table 1 presents the results of the elemental and ash analysis for the activated carbons obtained by chemical activation of coal from the Labin and Spitsbergen collieries using sodium hydroxide.

The symbols used in this work represent the following:

- LA—precursor (low-rank coal Labin) activated with sodium hydroxide at 600 °C in a nitrogen atmosphere for 45 min (weight ratio of activator:precursor, 2:1);
- SA—precursor (low-rank coal Spitsbergen) activated with sodium hydroxide at 600 °C in a nitrogen atmosphere for 45 min (weight ratio of activator:precursor, 2:1)

The data show that the activated carbon designated as LA contained the highest C^daf^ content, at 74.6 wt.%. Both of the tested activated carbons showed lower contents of the other four elements compared to the elemental carbon. The activated carbon from the Spitsbergen collier contained approximately 30% less carbon than the LA sample. The low elemental carbon content of the SA sample resulted in a high mineral content of up to 43.4 wt.%. This high ash content may cause pore clogging in the sample structure, hindering the reaction between the adsorbate and the adsorbent [17]. Furthermore, the ICP method was used to determine the weight percentage of certain elements in the ashes of the adsorbents. The LA activated carbon ash was found to contain a substantial amount of sulfur (3.342%). Additionally, the mineral substance obtained from the LA sample exhibited low levels of heavy metals, including Cu (0.012%), Cd (0.009%), Cr (0.010%), and Pb (0.008%). For the SA sample, the mineral matter contained 0.010% sulfur, 0.003% copper, 0.002% cadmium, 0.001% chromium, and 0.002% lead.

**Figure 1:**
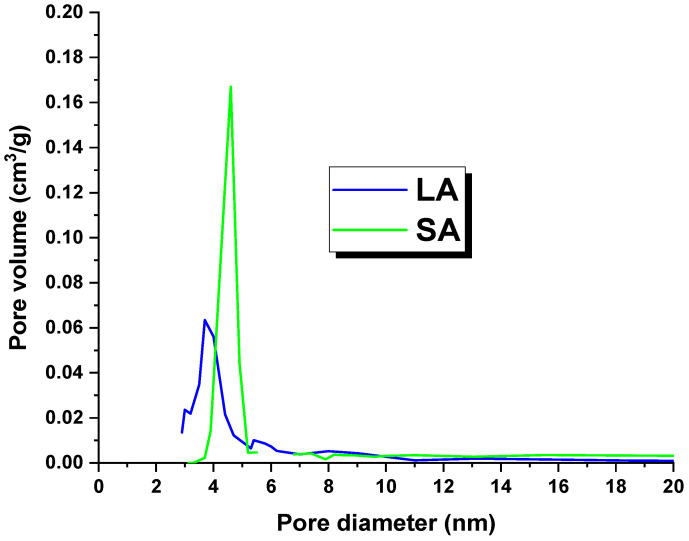
Pore distribution of the activated carbons.

Table 2 shows the textural parameters of the activated carbons. It is worth noting that the LA sample had a surface area of 520 m^2^/g, while the SA coal had a surface area of 138 m^2^/g. These surface areas were lower than those reported in the literature for lignite-derived activated carbons [18]. The possible reason for this issue could be the high concentration of mineral substances in the precursors used. These substances may have blocked or clogged the pores, which in turn prevented an effective reaction between the precursor and the activator [16]. An analysis of the data presented in Table 1 indicated that the adsorbents obtained were dominated by mesopores. This observation is further supported by Figure 1, which shows the pore size distribution of the LA and SA samples. Additionally, the total pore volume and micropore volume values suggested that the LA carbon contained more micropores in its structure.

**Figure 2:**
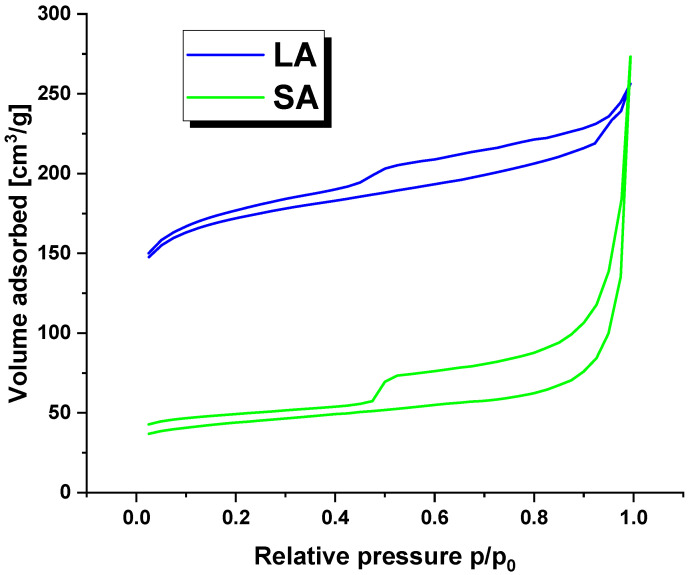
The nitrogen adsorption/desorption isotherms for activated carbons.

Figure 2 displays the nitrogen adsorption/desorption isotherms for the LA and SA samples. The curves follow a characteristic type IV isotherm pattern with a clear hysteresis loop, indicating the presence of mesopores in the activated carbon structure [19].

**Figure 3:**
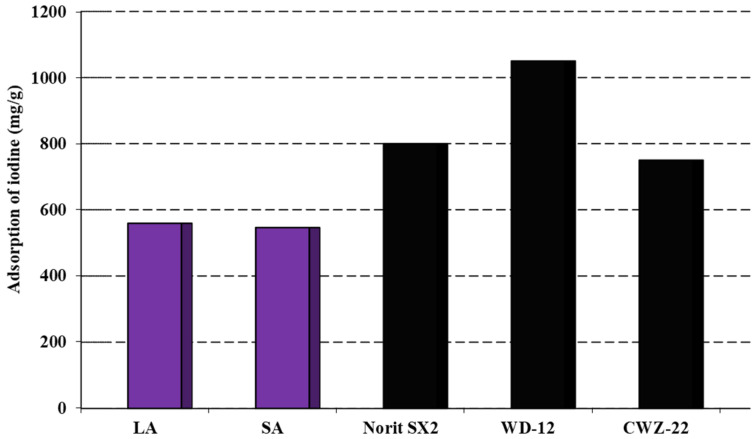
Adsorption of iodine on the activated carbons.

The analysis of the data presented in Figure 3 suggested that the obtained activated carbons had the ability to remove inorganic contaminants with sizes similar to the iodine molecule. From the results obtained, it can be concluded that these activated carbons showed similar sorption capacities towards aqueous iodine solutions. By analyzing the data in both Figure 3 and Table 2, it was concluded that the size of the specific surface area and the starting material influenced the amount of iodine adsorbed on the obtained carbon adsorbents. The most efficient adsorbent was found to be that produced through the chemical activation of coal sourced from the Labin colliers using sodium hydroxide. This coal exhibited a sorption capacity of 556 mg/g. The LA sample demonstrated a slightly lower sorption capacity of 546 mg/g. However, the activated carbons obtained had considerably lower sorption capacities for iodine when compared to commercially available carbons on the market. Carbons such as Norit SX2 and WD-12 possess a more developed specific surface in contrast to the LA and SA samples, which undoubtedly contributes to their sorption capacity [20]. Therefore, future research should concentrate on producing carbon adsorbents with significantly enhanced specific surface areas and porous structures.

Comprehending the pivotal acid–base characteristics is paramount in adsorption investigations of carbonaceous materials. This comprehension facilitates the anticipation of an activated carbon’s efficacy in adsorbing contaminants. Integral to this is the assessment of oxygen functional groups, which are capable of displaying either acidic or basic traits [21]. In this study, the quantification of surface functional groups across all carbon materials was conducted using the Boehm titration method. The results are outlined in Table 3. Additionally, the pH_pzc_ (point of zero charge) of the carbon adsorbents was determined. The surface of the LA carbon contained only acidic groups (1.01 mmol/g), whereas the carbon labelled SA contained only basic groups (1.30 mmol/g). The high basic nature of the SA sample was attributed to the presence of chromene- and pyrene-like groups, as well as a large amount of mineral matter deposited in the adsorbent’s pores. The acidity of the LA sample, however, may be attributed to the presence of a significant amount of sulfur groups. The pH_pzc_ values for the LA and SA samples were 7.4 and 6.0, respectively. In the adsorption studies, the cationic dye rhodamine B was used, indicating that the resulting activated carbons will efficiently remove the dye at pH values higher than the pH_pzc_ values of the samples. This is because of the stronger electrostatic attraction between the cationic dye and the negatively charged surface of the activated carbons [22].

### 2.2. Adsorption/Desorption Study

**Figure 4:**
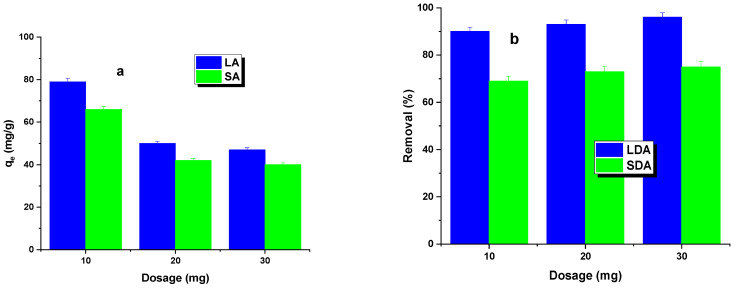
Effect of dosage (**a**) and adsorption efficiency (**b**) on Rhodamine B adsorption.

The first phase of the adsorption study investigated the effect of the activated carbon dosage on the dye removal efficiency. The results of these studies are shown in Figure 4. It was found that, as the dosage of the LA and SA samples increased, a decrease in the adsorption capacity was observed, while the percentage of dye removal increased. The sorption capacity decrease was more significant for LA carbon. Increasing the carbon dosage led to a larger contact area, resulting in more active sites capable of adsorbing the dye [23]. This led to a percentage increase in dye removal for both types of activated carbon. However, increasing the adsorbent dosage from 20 to 30 mg resulted in only a minimal increase in the dye removal efficiency, regardless of the type of activated carbon used. Therefore, an adsorbent dosage of 20 mg was adopted for further studies.

**Figure 5:**
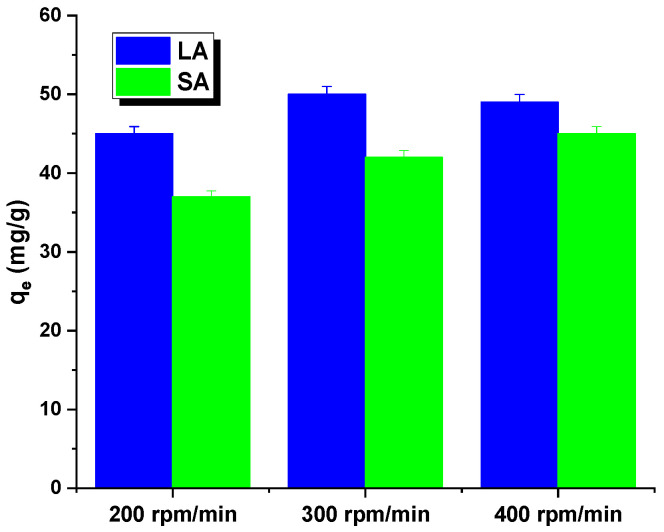
Effect of shaking speed on Rhodamine B adsorption by activated carbon.

The rate of shaking of the samples may have also impacted the interpretation of the results. Figure 5 shows that both the adsorbents had an identical relationship. The sorption capacity for the aqueous rhodamine B solution increased as the shaking speed increased from 200 to 300 rpm. This may have been due to a decrease in the thickness of the boundary layer around the carbon particles, caused by an increase in the mixing rate. While shaking the mixture, the activated carbon particles moved rapidly in the solution, leading to an increase in the concentration of rhodamine B in the vicinity of the adsorbent surfaces that potentially reached the total concentration. However, at shaking speeds exceeding 400 rpm, the diffusion rate decreased. This could have been due to the high shaking speed providing enough additional energy to break the newly formed bonds between rhodamine B and the activated carbon surface [16]. Further adsorption studies were conducted for the samples at a shaking speed of 300 rpm.

In the subsequent stages of the research, the sorption capacity of the activated carbons regarding the analyzed organic pollutant was assessed. The experimental sorption capacities of the utilized adsorbents were as follows: 117 mg/g for LA carbon and 61 mg/g for the SA sample (Table 4). Despite employing identical activation procedures for both initial materials, the resulting sorption capacities exhibited significant discrepancies, indicating the influence of various factors. An analysis of the data presented in Table 2 suggested that the specific surface area and the degree of porous structure development of the obtained adsorbents play a role in determining their sorption capacities. Notably, the LA sample, characterized by a higher S_BET_ value, demonstrated a superior sorption capacity compared to the SA adsorbent. Furthermore, the chemical properties of the activated carbons may have also contributed significantly to this disparity. The LA carbon presented an acidic surface character, while its counterpart exhibited a basic nature. In aqueous environments, acidic functional groups undergo dissociation, resulting in a negatively charged adsorbent surface, which is particularly favorable for the adsorption of cationic dyes such as rhodamine B. Consequently, the sorption capacity of the LA carbon surpassed that of the SA carbon by nearly double [24].

**Figure 6:**
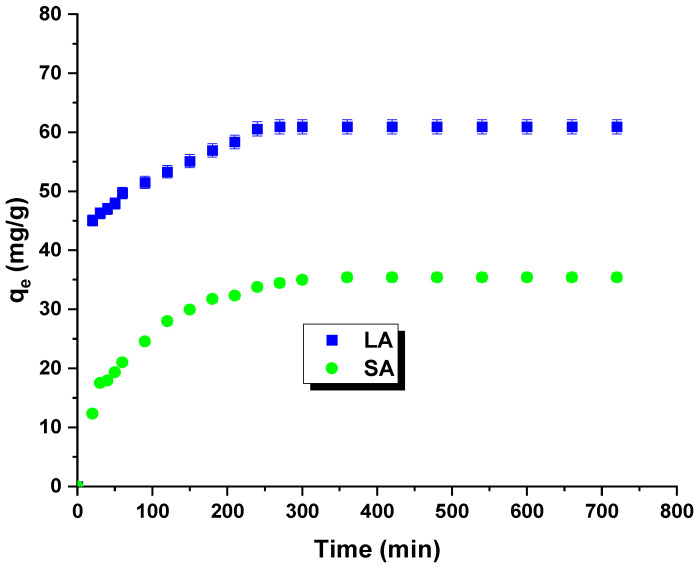
Effect of contact time, activated carbon, and rhodamine B on adsorption capacity.

Figure 6 shows the results of the kinetic studies. The isotherms allowed for the determination of the optimal time for the adsorption process. The time of formation of the adsorption equilibrium corresponds to the highest removal efficiency for rhodamine B. The curves indicate a rapid increase in the sorption capacity with contact time. The equilibrium state was achieved after 240 min. The maximum sorption capacity for the LA carbon was experimentally determined to be 60 mg/g, and for the SA sample, it was 35 mg/g. The initial increase in the sorption capacity can be attributed to the enhanced contact potential between the dye molecules and the synthesized adsorbents [25].

The aim of the kinetic studies was to identify a suitable model for characterizing the adsorption process. The experimental data were most accurately described by a pseudo-second-order model, which was confirmed by the high fit coefficient R^2^ value of 0.999 for both carbons (refer to Table 4). If the adsorption process adheres to pseudo-second-order kinetics, it suggests that chemical adsorption plays a crucial role in the overall process [26]. Additionally, the quantity of rhodamine B that was adsorbed, as calculated by this model, matched the experimental value of *q_t_* for the adsorbents [26]. However, the pseudo-first-order model and intramolecular diffusion yielded very low R^2^ values ranging from 0.567 to 0.897. Therefore, it is not recommended to use them to describe the adsorption kinetics of the impurity investigated for the obtained samples.

An important step in the realization of the adsorption process of an aqueous solution of rhodamine B on the adsorbents studied is the adjustment of the experimental data obtained to an appropriate adsorption isotherm model. The results presented are summarized in Table 5. An analysis of the parameters obtained showed that, regardless of the activated carbon used, the fit of the experimental data is best represented by the Langmuir model. The values of the correlation coefficient R^2^ for this model were 0.991 for the LA sample and 0.987 for SA, respectively. The Langmuir isotherm model assumes the theory of monolayer adsorption [27]. It is also worth noting that the sorption capacity factor q_m_ was close to the obtained experimental values of *q_e_*. Another important parameter determined for the Langmuir model was the dimensionless separation coefficient, the values of which indicate the favorable course of rhodamine B adsorption on the synthesized adsorbents. The last parameter of the model was the *K_L_* constant, which determines the affinity of the adsorbent for the pollutant to be removed. The higher the *K_L_* value, the greater the sorption capacity of the activated carbon towards the adsorbate. For the adsorbents used, the LA sample showed a higher affinity for rhodamine B, which also confirmed the achievement of a higher sorption capacity for this carbon. Significantly lower R^2^ values were obtained for the Freundlich, Temkin, and Dubinin–Radushkevich models compared to the results for the Langmuir isotherm. For this reason, it was presumed that these models do not play an important role in understanding the interaction between the adsorbent and the adsorbate.

**Figure 7:**
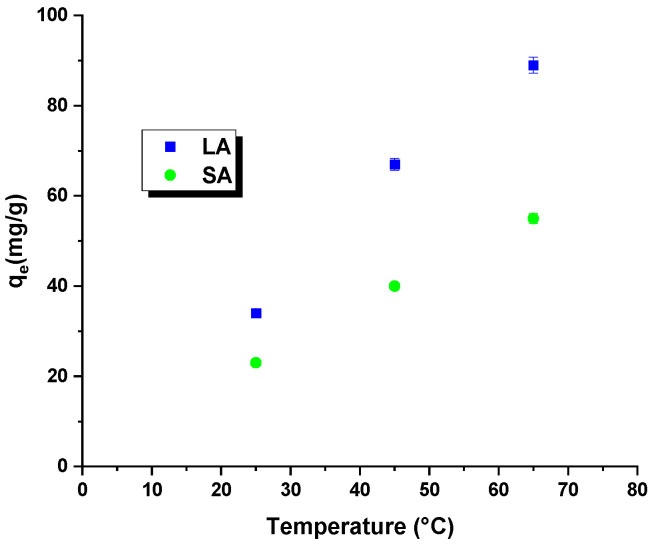
Effect of process temperature on rhodamine B removal efficiency.

Temperature is a crucial factor that affects the efficiency of removing harmful organic compounds through adsorption. Tests conducted at different temperatures help determine essential thermodynamic parameters, such as the change in enthalpy, entropy, and free energy. The analysis of the Figure 7 and Table 6 data indicated that increasing the process’s temperature improved the dye removal efficiency for both samples. Higher temperatures in the reaction system led to an increased mobility of the rhodamine B molecules. This resulted in a faster and more efficient interaction of these molecules with the surface of the activated carbons and their penetration into the pores of the adsorbent. Moreover, the free energy change was negative throughout the temperature range, confirming the spontaneous nature of the process due to an increase in the kinetic energy of the rhodamine B molecules. Regardless of the type of activated carbon used, the enthalpy change was positive, indicating the endothermic nature of the process. A further analysis of the Table 6 data revealed positive values for the entropy change. The entropy change (∆*S*^0^) of the LA carbon was measured to be 167.22 J/mol × K, whereas for the SA sample, it was 111.23 J/mol × K. These positive values indicate an increase in randomness at the interface between the activated carbon and the rhodamine B solution, resulting in a higher efficiency of dye removal [28].

**Figure 8:**
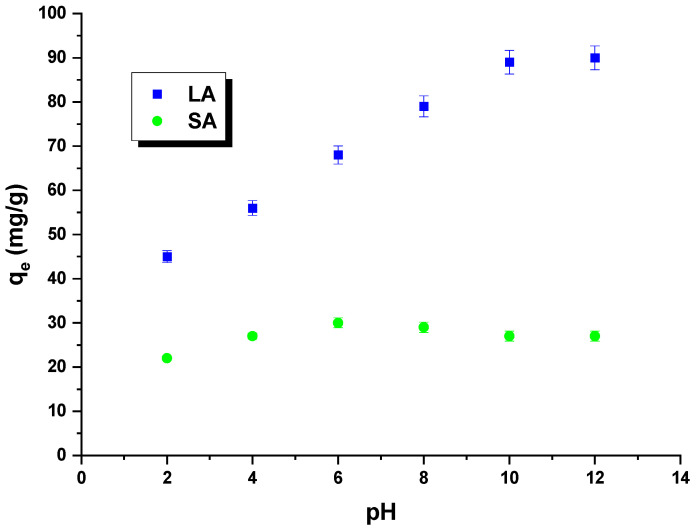
The impact of pH on rhodamine B adsorption.

Rhodamine B is an alkaline dye that exhibits different forms depending on the pH of the solution. At a pH below 3.5, the dye molecules are present in a monomeric form, while at a pH equal to or greater than 3.5, they assume a dimeric form. In this context, H^+^ ions can compete with dye cations, and OH^−^ groups can compete with dye anions. The impact of the pH of rhodamine B solutions on the sorption capacity was investigated for the obtained samples (Figure 8). The pH of the dye solution ranged from 2 to 12. It was observed that the quantity of rhodamine B adsorbed on the LA carbon increased as the pH of the dye solutions increased. This can be attributed to the electrostatic interactions between the negatively charged surface of the LA sample and the dye molecules. The sorption capacity of SA carbon was found to only slightly increase when the pH of the dye solution was increased from 2 to 6. However, when the pH was further increased from 6 to 12, the sorption capacity decreased. It is important to note that the changes in sorption capacity over the studied pH range were minimal. This was likely due to the fact that the carbon had only acidic groupings on its surface, which did not interact with the dye molecules. As a result, this sample exhibited low sorption capacities [29].

The process of adsorption relies on the interaction between the adsorbate and the adsorbent. Synthesized activated carbons possess active sites on their surface that enable rhodamine B molecules to enter the pores. Additionally, there may be interactions such as π-π interactions, hydrogen bonds, and electrostatic interactions between the adsorbate and the adsorbent. The adsorbate’s functional groups are expected to form hydrogen or electrostatic bonds with the functional groups on the activated carbon surface. The π-π interactions occur due to the presence of rhodamine B benzene and aromatic rings on the activated carbon surface [29].

The desorption of rhodamine B from the surface of the activated carbons was investigated (Table 7). The desorption efficiencies ranged from 7% to 82%. The highest desorption efficiency was observed for 0.1 M HCl. The order of desorption efficiency was as follows: HCl > KOH > H_2_O > CH_3_COOH. A hydrochloric acid solution was the most effective eluent, as the acidic environment displaced the dye molecules from their binding sites with protons [30]. This study demonstrated the potential for regenerating the synthesized adsorbents. However, to enhance the efficiency of the desorption process, it is advisable to select alternative eluents.

Table 8 summarizes the maximum adsorption capacities of various adsorbents for rhodamine B. A variety of carbon adsorbents have been deliberately juxtaposed to demonstrate that adsorption studies on rhodamine B are widespread. The adsorbent derived from tea leaves retained 53.2 mg/g of the dye [30]. The adsorption mechanism of rhodamine B on composite materials based on graphene oxide and beta zeolite was also investigated [31]. This composite exhibited a slightly better ability to remove the dye from aqueous solutions, at 64.47 mg/g. The presented results were significantly lower than the sorption capacity of the LA sample, but comparable to the results obtained for SA carbon. Table 8 shows that the adsorbents described in other studies [32,33] had higher sorption capacities than those presented in this study. This study investigated the adsorption of rhodamine B on activated carbons from the Labin collieries and Spitsbergen. The adsorbents were obtained by the chemical activation of the precursors with potassium hydroxide. The sorption capacities of the activated carbons from both sources were found to be higher than those of the LA and SA samples. The samples described in [16] exhibited higher sorption capacities, which could be attributed to their better-developed specific surface area and porous structure, as well as the use of a more reactive activator.

## 3. Materials and Methods

### 3.1. Materials

The following coals were the starting material for the studies presented in this thesis: (1) lignite from the Labin colliery and (2) hard coal from the Spitsbergen colliery. The raw coals were crushed and then sieved through a 0.09 mm mesh sieve. Physicochemical and sorption tests were carried out on the demineralized coals [16]. The rhodamine B used for the analysis was obtained from Merck (Darmstadt, Germany), while the remaining chemicals were acquired from Sigma-Aldrich (Burlington, MA, USA) and were of analytical grade.

### 3.2. Preparation of Activated Carbons

The demineralized carbons were activated in a tube furnace using conventional heating. The process was conducted under a nitrogen atmosphere with a flow rate of 330 mL/min at 600 °C (5 °C/min) for 45 min using sodium hydroxide as the activator at a weight ratio of 2:1 to the precursor. The adsorbents produced were washed with 5% hydrochloric acid to eliminate excess sodium hydroxide. Subsequently, they were flooded with a HCl solution and boiled under a reflux condenser. In the following step, the coals were washed with distilled water until a neutral reaction was achieved and then dried at 110 °C.

### 3.3. Activated Carbon Characterization

The elemental composition of the activated carbons was analyzed using a Thermo Scientific FLASH 2000 Elemental Analyzer (Elementar Analysensysteme GmbH, Langenselbold, Germany). To evaluate the ash content, the samples were combusted in a microwave muffle furnace at 815 °C for 60 min. The total concentrations of the ash elements were determined by high-resolution optical emission spectrometry with inductively coupled plasma (ICP hrOES). A PlasmaQuant PQ 9000 Elite spectrometer (Analytic, Jena, Germany) was used. The measurement procedure is described in our earlier work [34]. The concentration of oxygen functional groups on the surface of the activated carbons was assessed using the Boehm method [16]. The textural characteristics of the activated carbons were determined using a low-temperature nitrogen sorption/desorption analysis performed with the Quantachrome Autosorb IQ apparatus (Boynton Beach, FL, USA). The methodology used in this research is described in [16]. Additionally, an iodine adsorption analysis was conducted for the obtained biochars [16].

### 3.4. Adsorption Study

An aqueous solution of rhodamine B was used in the adsorption studies. A dye stock solution of 1000 mg/dm^3^ was prepared, from which working solutions were prepared in the concentration range from 5 to 50 mg/L. At the start of the adsorption studies, we investigated the influence of the adsorbent mass on the sorption capacities towards the dye under study. We used three different weights of activated carbon: 0.010 g, 0.020 g, and 0.030 g. Next, we examined the effect of the shaking rate on the sorption capacities of the tested activated carbons. The tests were conducted at three different shaking speeds: 200, 300, and 400 rpm/min. The mixtures were shaken for 24 h on a shaker in each case (Heidolph, Schwabach, Germany). Then, 50 cm^3^ of a 25 mg/dm^3^ dye solution was added to the carbons. The effect of the activated carbon mass on the dye removal efficiency was assessed using shaking at 300 rpm/min. After shaking, the solid samples were separated from the solution using a laboratory centrifuge. The sorption capacity of the carbon adsorbents was determined by spectrophotometric measurements.

For further adsorption studies, a mass of 0.020 g of activated carbon and a shaking rate of 300 rpm/min were assumed based on the measurements. The experimental procedure consisted of adding 0.020 g of activated carbon to 200 cm^3^ glass bottles, followed by pouring 50 cm^3^ of a dye solution with a specified concentration. The activated carbon was then shaken on a shaker for 24 h at 300 rpm/min and room temperature. After the process, the activated carbon sample was separated from the solution using a laboratory centrifuge. The concentration of the dye in the solution was determined spectrophotometrically at a maximum absorption wavelength of 554 nm using a Carry 100 Bio spectrophotometer (Agilent, Santa Clara, CA, USA). All experiments were repeated twice. The sorption capacities of the activated carbons were calculated using the corresponding equation:

(1) $$q_{e}=\frac{C_{0}-C_{e}}{m}\times V$$

where: *C*_0_—initial concentration of the rhodamine B solution (mg/L); *C_e_*—concentration of rhodmaine B remaining in the solution at equilibrium (mg/L); *m*—weight of the adsorbent (g); and *V*—volume of the rhodamine B solution (L).

The optimization of contact time between the adsorbate and the adsorbent was performed using 50 cm^3^ of a 25 mg/L dye solution and an adsorbent mass of 0.020 g. The shaking speed was kept at 300 rpm for 24 h. The results of this study allowed for the determination of kinetic parameters specific to three models: pseudo-first-order, pseudo-second-order, and intraparticle diffusion.

The pseudo-first-order model [35] assumes that the removal rate of rhodamine B is related to the number of unoccupied adsorption centers. This is expressed by the following equation:

(2) $$\log(q_{e}-q_{t})=logq_{e}-\frac{k_{1}}{2303}$$

where *k*_1_—the kinetic constant of the pseudo-first-order model (1/min).

In contrast, the pseudo-second-order model [35] is based on the assumption that a chemisorption process occurs between the adsorbent and the adsorbate:

(3) $$\frac{t}{q_{t}}=\frac{1}{k_{2}q_{e^{2}}}+\frac{t}{q_{e}}$$

where *k*_2_—the second-order rate constant (g/mol × min).

The intraparticle diffusion model [36] is defined by the following equation:

(4) $$q_{t}={K_{IPD}}^{1/2}+C$$

where: *C*—boundary layer thickness (mg/g) and *K_IPD_*—intraparticle diffusion rate constant (mg/g × min^1/2^).

This study compared experimental data with the parameters calculated for the linear forms of four models: the Langmuir, Freundlich, Temkin, and Dubinin–Radushkevich models. The Langmuir model [37] assumes that the dye adsorption process occurs uniformly on the carbon surface:

(5) $$\frac{C_{e}}{q_{e}}=\frac{1}{K_{L}\times q_{max}}+\frac{C_{e}}{q_{max}}$$

where: *K_L_*—Langmuir constant (L/mg) and *q_max_*—adsorption capacity of the monolayer (mg/g).

The Freundlich isotherm model [38] refers to multilayer adsorption:

(6) $$logq_{e}=logK_{F}+\frac{1}{n}\times logC_{e}$$

where: *K_F_*—Freundlich constant (mg/g(L/mg)^1/n^) and 1/*n*—constant related to the affinity of the adsorbate for the adsorbent.

The Temkin model [39] explains the linear relationship between the coverage area of activated carbon and the heat of adsorption:

(7) $$lnq_{e}=BlnA_{T}+BlnC_{e}$$

where: *B*—constant related to the heat of adsorption (J/mol) and *A_T_*—an empirical constant related to the maximum binding energy (L/mg).

This study employed the Dubinin–Radushkevich model [40] as its fourth model. This model is based on a pore-filling mechanism and assumes a heterogeneous adsorbent surface with a varying adsorption potential.

(8) $$lnq_{e}=lnq_{max}-\beta \varepsilon^{2}$$

where: *ε*—the Polanyi potential and *β*—a constant related to the adsorption energy (mol^2^/kJ^2^).

To describe the thermodynamics of the adsorption process of rhodamine B on the resulting activated carbons [41], thermodynamic parameters such as the enthalpy (Δ*H*^0^, kJ/mol), entropy (Δ*S*^0^, kJ/K × mol), and Gibbs free energy (Δ*G*^0^, kJ/mol) were determined using the following equations:

(9) $$∆G^{0}=-RTlnK_{d}$$

(10) $$∆G^{0}=∆H^{0}-T\Delta S^{0}$$

where: Δ*G*^0^—Gibbs free energy, *R*—universal constant (8314 J/mol × K), *T*—temperature (K), *K_d_*—thermodynamic equilibrium constant, Δ*H*^0^—enthalpy change, and Δ*S*^0^—entropy change.

The influence of the pH of the rhodamine B solution on the adsorption capacity of the activated carbons was assessed across a pH range of 2 to 12. The measurements were conducted over a period of 24 h, using 0.020 g of the sample and 50 mL of a dye solution with a concentration of 25 mg/L.

### 3.5. Desorption Study

A total of 1 g of activated carbon was mixed with 50 mL of a rhodamine B solution with a concentration of 25 mg/L for 8 to 24 h. Following the attainment of an adsorption equilibrium, the activated carbon was drained and rinsed with distilled water to eliminate any residual adsorbate. Subsequently, the adsorbents were air-dried. The desorption solutions comprised 0.1 M hydrochloric acid, 0.1 M potassium hydroxide solution, water, and 0.1 M acetic acid. The desorption process lasted for 24 h.

## 4. Conclusions

This study concluded the following: (1) the activated LA carbon exhibited the most well-developed specific surface area and porous structure; (2) the acid–base properties of the activated carbons were primarily influenced by the type of precursor used; (3) the LA sample demonstrated a notably acidic surface character, while the SA carbon contained only basic groupings in its structure; (4) the sorption capacities of the carbons obtained in this study towards iodine ranged from 546 to 556 mg/g; (5) the experimental sorption capacities of the activated carbons towards an aqueous solution of rhodamine B were found to be in the range of 61–117 mg/g; (6) the Langmuir isotherm was found to best describe the adsorption process of the dye molecules on the surface of the activated carbons; (7) the pseudo-second-order model yielded the highest correlation coefficient R^2^ values; (8) the thermodynamic analysis indicated that the adsorption of rhodamine B was an endothermic process and occurred spontaneously; and (9) the desorption studies indicated that the 0.1 M HCl solution was the most effective eluent.

## Figures and Tables

**Table 1:** The elemental composition and content of ash of the activated carbons (wt.%).

Sample	C^daf^	H^daf^	N^daf^	S^daf^	O^daf^ *	Ash
LA	74.6	1.8	1.0	6.2	16.4	9.6
SA	49.4	1.0	0.3	0.6	48.7	43.4

dry—dry ash-free basis, *—by difference, method error ≤ 0.3%.

**Table 2:** Textural parameters of the activated carbons.

Sample	Surface Area * (m^2^/g)	Micropore Area (m^2^/g)	Total Pore Volume (cm^3^/g)	Micropore Volume (cm^3^/g)	Average Pore Diameter (nm)
LA	520	198	0.40	0.14	3.05
SA	138	57	0.42	0.03	12.32

* Error range between 2 and 5%.

**Table 3:** Acid-base properties of the activated carbons.

Sample	Acidic Groups * (mmol/g)	Basic Groups * (mmol/g)	pH_pzc_ *
LA	1.01	0.00	7.4
SA	0.00	1.30	6.0

* Arithmetic mean of the two determinations.

**Table 4:** Kinetic parameters determined using pseudo-first-order, pseudo-second-order, and intraparticle diffusion models.

Models	Parameters	Rhodamine B	
		LA	SA
	*q_t_* (mg/g)	60	35
pseudo-first-order	R^2^	0.897	0.789
	*k*_1_ (1/min)	8.67 × 10^−3^	2.41 × 10^−2^
	q_e,cal_ (mg/g)	16	10
pseudo-second-order	R^2^	0.999	0.999
	*k*_2_ (g/mg × min)	1.44 × 10^−3^	1.08 × 10^−3^
	q_e,cal_ (mg/g)	59	34
intraparticle diffusion	R^2^	0.667	0.567
	*k_IPD_* (mg/g × min^1/2^)	2.989	2.545
	X (mg/g)	29	19

**Table 5:** Isotherm study of rhodamine B.

Isotherms	Parameters	Rhodamine B	
		LA	SA
	*q_e_* [mg/g]	117	61
Langmuir	R^2^	0.991	0.987
	*q_max_* [mg/g]	119	63
	*K_L_* (L/mg)	0.071	0.052
Freundlich	R^2^	0.902	0.876
	*K_F_* (mg/g(L/mg)^1/n^)	112.33	34.67
	1/n	0.189	0.345
Temkin	R^2^	0.508	0.604
	*B* (J/mol)	67.98	123.89
	*A_T_* (L/mg)	15.45	5.23
Dubinin–Radushkevich	R^2^	0.899	0.845
	*q_max_* (mg/g)	110	57
	*E* (kJ/mol)	3.897	1.678

**Table 6:** Thermodynamic parameters of rhodamine B adsorption.

Sample	Temperature [°C]	Δ*G*^0^ (kJ/mol)	Δ*H*^0^ (kJ/mol)	Δ*S*^0^ (J/mol × K)
LA	25	−9.89	42.56	167.22
	45	−10.89		
	65	−12.67		
SA	25	−4.56	30.45	111.23
	45	−8.89		
	65	−10.89		

**Table 7:** Regeneration of activated carbons by different eluents (%).

Sample	H_2_O	KOH	HCl	CH_3_COOH
LA	56	41	82	13
SA	43	32	79	7

**Table 8:** Rhodamine B maximum adsorption capacity of selected adsorbents.

Adsorbent	Adsorption Capacity (mg/g)	References
Adsorbent from black tea leaves	53.2	[30]
Graphene oxide/beta zeolite composite	64.47	[31]
Activated carbon for white sugar	123.46	[32]
Gelatin/activated carbon composite beads	256.41	[33]
LA	119	This study
SA	63	This study
Labin_KOH_	175	[16]
Spitsbergen_KOH_	82	[16]

## Data Availability

The data are contained within the article.
